# Study of the Biotransformation of Tongmai Formula by Human Intestinal Flora and Its Intestinal Permeability across the Caco-2 Cell Monolayer

**DOI:** 10.3390/molecules201018704

**Published:** 2015-10-15

**Authors:** Shuai Wu, Wei Xu, Fu-Rong Wang, Xiu-Wei Yang

**Affiliations:** State Key Laboratory of Natural and Biomimetic Drugs, Department of Natural Medicines, School of Pharmaceutical Sciences, Peking University Health Science Center, Peking University, No. 38, Xueyuan Road, Haidian District, Beijing 100191, China; E-Mails: 4580039@163.com (S.W.); high-xu@163.com (W.X.); wangfurong1983@gmail.com (F.-R.W.)

**Keywords:** tongmai formula, isoflavone, HPLC, traditional Chinese medicine, human intestinal flora, biotransformation, human Caco-2 cells monolayer

## Abstract

Tongmai formula (TMF) is a well-known Chinese medicinal preparation that contains isoflavones as its major bioactive constituents. As traditional Chinese medicines (TCMs) are usually used by oral administration, their fate inside the intestinal lumen, including their biotransformation by human intestinal flora (HIF) and intestinal absorption deserves study. In this work TMF extract was incubated with human intestinal bacteria under anaerobic conditions and the changes in the twelve main constituents of TMF were then investigated. Their intestinal permeabilities, *i.e.*, the transport capability across the intestinal brush border were investigated with a human colon carcinoma cell line (Caco-2) cell monolayer model to predict the absorption mechanism. Meanwhile, rapid HPLC-DAD methods were established for the assay. According to the biotransformation curves of the twelve constituents and the permeability coefficients, the intestinal absorption capacity of the typical compounds was elevated from the levels of 10^−7^ cm/s to 10^−5^ cm/s from those of the original compounds in TMF. Among them the main isoflavone glycosides puerarin (**4**), mirificin (**6**) and daidzin (**7**) were transformed into the same aglycone, daidzein (**10**). Therefore it was predicted that the aglycone compounds might be the real active ingredients in TMF. The models used can represent a novel path for the TCM studies.

## 1. Introduction

Tongmai formula (TMF) is a popular compound prescription including three well-known traditional Chinese medicines (TCMs), namely Puerariae Lobatae Radix [roots of *Pueraria lobata* (Willd.) Ohwi, Family Leguminosae], Salviae Miltiorrhizae Radix (roots of *Salvia miltiorrhiza* Bge., Family Labiatae) and Chuanxiong Rhizoma (rhizomes of *Ligusticum chuanxiong* Hort., Family Umbelliferae) in a weight ratio of 1:1:1. TMF is widely used for the treatment of ischemic cerebrovascular and cardiovascular diseases, such as myocardial infarction and atherosclerosis, for lowering blood lipids, or improving blood viscosity [1,2,3]. Previous studies on TMF were mainly focused on its chemical constituents and 34 compounds were isolated from the water decoction of TMF by our group [4]. Reports also established the chemical constituent fingerprint of TMF and quantitative analysis of the major chemical constituents for quality control purposes [5,6,7,8]. These researches demonstrated that isoflavones (especially puerarin and daidzein) and their derivatives were the major bioactive constituents of TMF.

Conventionally TCMs are administered orally. In the intestine the constituents of TCM first meet the intestinal bacteria, a vast gut microecological community. Studies on the bacterial flora in the alimentary canals of mammalia have proved that 99% of the bacteria are anaerobic. The classification and distribution of anaerobes in the digestive tract have also been brought to light, and the metabolizing abilities of intestinal bacteria and biochemical interactions between a host and its intestinal flora have become the object of extensive research. As we all know, the intestinal bacteria play a significant role in the biotransformation of endogenous and xenobiotic substances, including diverse drug molecules [9,10]. However, *in vivo*/*in vitro* research on the biotransformation of TMF is still a neglected topic, due to its complicated multicomponent nature and the difficulty of preparing biotransformation products, so a systematic study on the biotransformation of TMF is necessary. Biotransformation studies can also assist in understanding the mechanism of the therapeutic benefits or adverse effects of drugs [11,12]. Therefore, the human intestinal flora (HIF) model *in vitro* was used to investigate the biotransformation of TMF.

**Figure 1:**
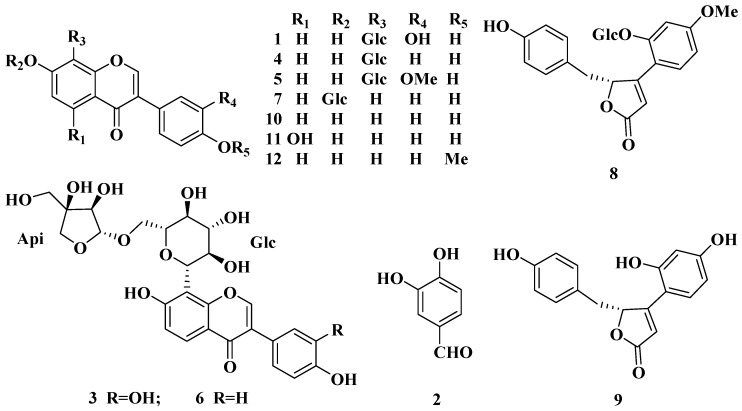
Chemical structures of the 12 main compounds from Tongmai Formula: 3′-hydroxypuerarin (**1**), protocatechuic aldehyde (**2**), 3′-hydroxymirificin (**3**), puerarin (**4**), 3′-methoxypuerarin (**5**), mirificin (**6**), daidzin (**7**), (±)-puerol B-2″-*O*-glucopyranoside (**8**), puerol A (**9**), daidzein (**10**), genistein (**11**), and formononetin (**12**).

In this research, a simultaneous determination of the major twelve constituents of TMF transformed by HIF (3′-hydroxypuerarin (**1**), protocatechuic aldehyde (**2**), 3′-hydroxymirificin (**3**), puerarin (**4**), 3′-methoxypuerarin (**5**), mirificin (**6**), daidzin (**7**), (±)-puerol B-2″-*O*-glucopyranoside (**8**), puerol A (**9**), daidzein (**10**), genistein (**11**), and formononetin (**12**)) [4,13] (Figure 1) was developed using a fully validated and economical high performance liquid chromatography (HPLC) method. Then the time course of the twelve main constituents in TMF during the biotransformation by HIF was established, which should help clarify the biotransformation and pharmacokinetics properties as well as pharmacological mechanism of TMF.

After the biotransformation by HIF, the resulting constituents will face a barrier before entering the body, the intestinal epithelium. Human colon carcinoma cell line (Caco-2) cell monolayers exhibit enterocyte-like characteristics and provide a mature model simulating the human small intestinal epithelium. In the recent years, this monolayer model has been widely used as an *in vitro* model of absorption across intestinal epithelial cells, that is available and practical for screening the intestinal permeability of active constituents [14]. In the present study, we focused on the intestinal permeability in the human Caco-2 cell monolayer model of the isoflavones of TMF and their analogues (compounds **1** and **3**–**12**), and their structure–permeability relationships were also discussed.

## 2. Results and Discussion

### 2.1. Methodological Validation of the Quantitative Analysis for the Twelve Constituents of TMF

**Figure 2:**
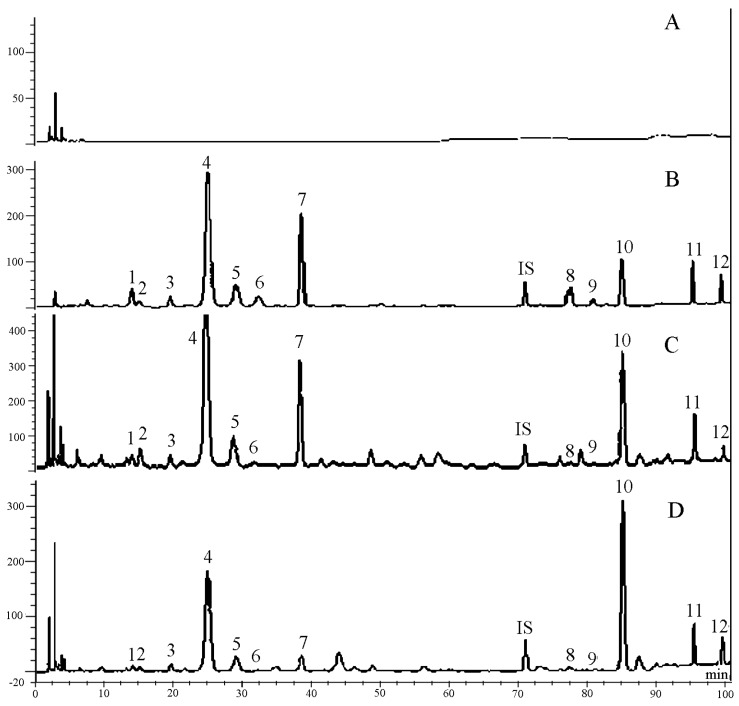
Typical chromatograms of the twelve constituents of TMF and the internal standard (I.S.) in the biotransformation experiments: (**A**) blank incubation solution; (**B**) standard solutions of the twelve constituents and I.S.; (**C**) the medium solution obtained at 0.5 h after biotransformation by HIF; (**D**) the medium solution obtained at 48 h after biotransformation by HIF.

Chromatographic peaks of the twelve constituents and naringin used as an internal standard (I.S.) showed very good resolutions. The average retention times for the twelve constituents and the I.S. were 14.2, 15.2, 19.8, 25.2, 29.3, 32.5, 38.7, 77.8, 81.0, 85.1, 95.4, 99.5, and 71.1 min, respectively. The chromatographic profiles of the blank and heat-inactivated HIF were investigated, and no significant interferences were observed at the retention times of the twelve constituents and I.S. (Figure 2).

The tests of linearity, lower limits of detection (LLODs) and lower limits of quantitation (LLOQs), precision, repeatability, stability, and recovery were determined by using the optimized method of HPLC, which are shown in Supplementary Tables S1 and S2 (see Supplementary Materials). All of the correlation coefficient (*r*) values were above 0.99, which indicated a good linear correlation. The LLODs and LLOQs were 0.02–0.65 μg/mL and 0.07–1.96 μg/mL, respectively. The intra- and inter-day accuracies ranged from 91.29% to 109.54%, and the relative standard deviations (RSDs) of intra-day and inter-day precisions were below 12.67% and 11.88%, respectively. The extraction recovery rates ranged from 62.80% to 86.95% with RSD values less than 13.24%. The analytes were found to be stable at room temperature for 24 h as the accuracies of stability were in range of 97.15% to 106.26%. All these values were found to be in an acceptable range, indicating that the method was accurate, reproducible and reliable for assessing the quality of TMF according to the guidelines of the United State Food and Drug Administration (FDA) for bioanalytical method validation [15].

### 2.2. Time Course of the Twelve Constituents

**Figure 3:**
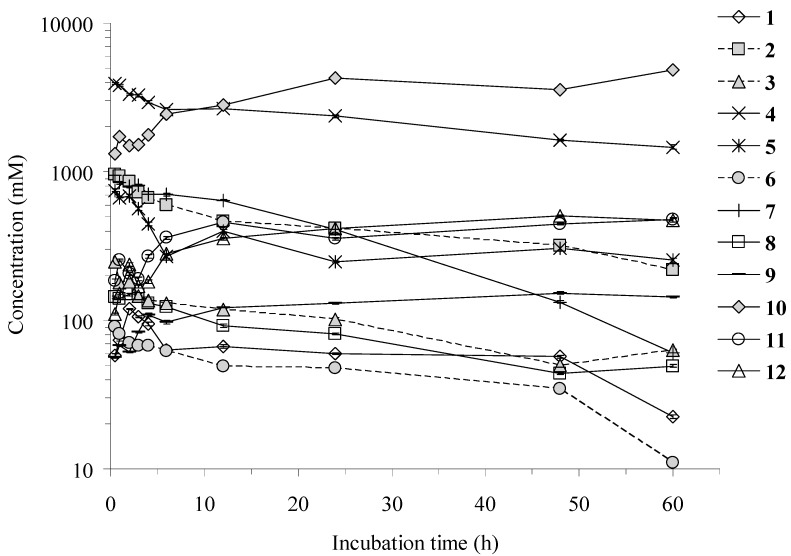
Time course of the twelve constituents of TMF by HIF (*n* = 6, mean ± SD).

The method developed was successfully applied to the studies of twelve constituents of TMF by HIF. As shown in Figure 3, the contents of the mirificin (**6**) and daidzin (**7**) were reduced by 10-fold after 60 h incubation by HIF, suggesting that the two compounds were easily transformed by HIF. At the same time, the content of the four aglycone compounds, puerol A (**9**), daidzein (**10**), genistein (**11**) and formononetin (**12**), increased more than 3-fold. At the early stage (2 h) of the 60 h incubation, mirificin (**6**) decreased fast, but the rate of disappearance of puerarin (**4**) was slow down at 2 h, suggesting that mirificin (**6**) was converted to puerarin (**4**) by HIF. Daidzin (**7**) was almost completely converted into daidzein (**10**) during the 60 h incubation by HIF, suggesting that daidzin (**7**) was a pro-drug of daidzein (**10**). However, daidzein (**10**) began to decrease suddenly at 2 h and 48 h, suggesting that daidzein (**10**) was further converted to other product(s) and itself might be a biotransformation intermediate. After the process of deglycosylation, demethoxylation and hydroxylation, (±)-puerol B-2″-*O*-glucopyranoside (**8**) could theoretically be converted into puerol A (**9**), suggesting that the puerol glycosides were pro-drugs of puerol aglycones. Genistein (**11**) and formononetin (**12**) are isoflavone aglycones (their glycosides are genistin and ononin) and showed an increased growth in the time curves, suggesting that they might be biotransformation intermediates. At the early stage (4 h) of incubation, 3′-hydroxymirificin (**3**) quickly reduced to nearly 25%, and 3′-hydroxypuerarin (**1**) reached a maximum concentration at 2 h, but decreased sharply after prolonged incubation, suggesting that 3′-hydroxymirificin (**3**) was a pro-drug of 3′-hydroxypuerarin (**1**). The contents of protocatechuic aldehyde (**2**) and 3′-methoxypuerarin (**5**) were reduced to nearly 50% at 60 h, suggesting that they were further transformed or incorporated into the bacterial cells. In conclusion, 3′-hydroxymirificin (**3**), mirificin (**6**), daidzin (**7**), and (±)-puerol B-2″-*O*-glucopyranoside (**8**) might be pro-drugs of 3′-hydroxypuerarin (**1**), puerarin (**4**), daidzein (**10**) and puerol A (**9**), respectively, and genistin and ononin might be the pro-drugs of genistein (**11**) and formononetin (**12**), respectively. From the perspective of growth and decline, compounds **1**–**8** were consumed and **10**–**12** were the products.

### 2.3. Transport of the Eleven Compounds across the Caco-2 Cell Monolayer

The linearity, precision, accuracy, and stability recovery of the HPLC methods were all validated for the assay (see Supplementary Materials Tables S3 and S4). To validate the Caco-2 cell monolayer system, apparent permeability coefficient (*P*_app_) values of propranolol, a well-transported marker by passive diffusion, and atenolol, a poor-transported marker by passive diffusion [16], from the apical (AP) to basolateral (BL) chamber across the Caco-2 monolayer were determined as 3.37 × 10^−5^ cm/s and 6.17 × 10^−7^ cm/s, respectively, which were comparable to the report [17].

The bidirectional *P*_app_ values of the eleven compounds have been summarized in Table 1. In general, well-absorbed drugs were found to have high *P*_app_ (>1.0 × 10^−5^ cm/s), moderately absorbed drugs were found to have a *P*_app_ value of 1–10 × 10^−6^ cm/s, whereas poor-absorbed drugs had low *P*_app_ (<1.0 × 10^−6^ cm/s) in the Caco-2 cell monolayer model [18]. The *P*_app_ values of isoflavone aglycones such as **10**, **11** and **12** were at a level of 10^−5^ cm/s and similar to that of propranolol, so they were assigned to the well-absorbed compound group. The *P*_app_ values of the isoflavone glycosides such as puerarin (**4**) and daidzin (**7**) both belonged to a level of 10^−7^ cm/s, two orders of magnitude lower than those of the isoflavone aglycones and therefore compounds **4** and **7** could be thought as poorly absorbed compounds. The *P*_app_ value of the aglycone daidzein (**10**) was much higher than that of its glycoside daidzin (**7**), suggesting that daidzein was taken up and transported more effectively than daidzin. It has been reported that the inhibitory effects of daidzein (**10**) on lipopolysaccharide-induced nitric oxide production in RAW 264.7 murine macrophages cells was stronger than that of daidzin [19].

**Table 1:** Bidirectional *P*_app_ values of the eleven compounds of TMF in the Caco-2 model.

Compound	*P*_app AP→BL_ (×10^−6^, cm/s)	*P*_app BL→AP_ (×10^−6^, cm/s)	*P*_app BL→AP_/*P*_app AP→BL_	LogD (pH 7.4)
**1**	0.45 ± 0.02	0.29 ± 0.01	0.64	–1.02
**3**	0.97 ± 0.01	0.53 ± 0.01	0.55	–1.61
**4**	0.68 ± 0.02	0.41 ± 0.01	0.60	–0.81
**5**	0.75 ± 0.09	0.47 ± 0.03	0.63	–0.69
**6**	0.29 ± 0.01	0.23 ± 0.01	0.79	–0.94
**7**	0.40 ± 0.01	0.37 ± 0.01	0.92	0.23
**8**	1.29 ± 0.28	1.03 ± 0.09	0.80	0.28
**9**	6.27 ± 0.81	8.30 ± 1.60	1.32	2.09
**10**	25.51 ± 0.34	26.22 ± 0.01	1.03	2.51
**11**	32.41 ± 3.86	37.70 ± 1.93	1.16	1.65
**12**	30.64 ± 0.07	26.53 ± 1.10	0.87	2.92

*P*_app_ _AP→BL_: Transport of the compounds from the apical to the basolateral side; *P*_app_ _BL→AP_: from the basolateral to the apical side; *P*_app_ _BL→AP_/*P*_app_ _AP→BL_: the ratio of *P*_app_ _BL→AP_/*P*_app_ _AP→BL_. The concentration of all compounds was 50 μM. The incubation time was up to 90 min. Data are means ± S.D. (*n* = 3).

The *P*_app_ value of puerol aglycone (**9**) was higher than that of puerol glycoside (**8**), though they were at a same level of 10^−6^ cm/s, which may be attributed to the extra methoxylation of **8** making it more lipophilic and resulting in a larger *P*_app_ value despite the glycosylation.

There was no indication of efflux or active transport because the ratios of *P*_app BL→AP_/*P*_app AP→BL_ for eleven compounds were between 0.55–1.32 according to the net efflux criterion proposed by the FDA Guidance (Table 1). This result suggests that passive diffusion is the main transport mechanism of all eleven compounds.

### 2.4. Mass Balance of the Eleven Compounds in the Caco-2 Cell Model

In order to check the mass balance, the recovery of each compound at the end of transport experiment was determined as the total amount in both AP and BL sides of the Caco-2 cells monolayer model. The recoveries of the eleven compounds were from 76% to 102% in both bidirectional transport studies as shown in Table 2, which suggests a high stability during the transport across the intestinal barrier.

**Table 2:** Total recoveries of the eleven compounds of TMF in the Caco-2 model.

Compound	Total Recovery (%)	
	AP→BL	BL→AP
**1**	98.08	94.43
**3**	100.61	98.28
**4**	96.62	95.70
**5**	94.62	86.15
**6**	99.42	98.51
**7**	99.75	98.55
**8**	77.01	91.42
**9**	86.05	90.87
**10**	93.44	100.35
**11**	86.72	75.68
**12**	101.82	97.99

The incubation time was up to 90 min. Data are means (*n* = 3).

### 2.5. Structure-Intestinal Permeability Relationship

**Figure 4:**
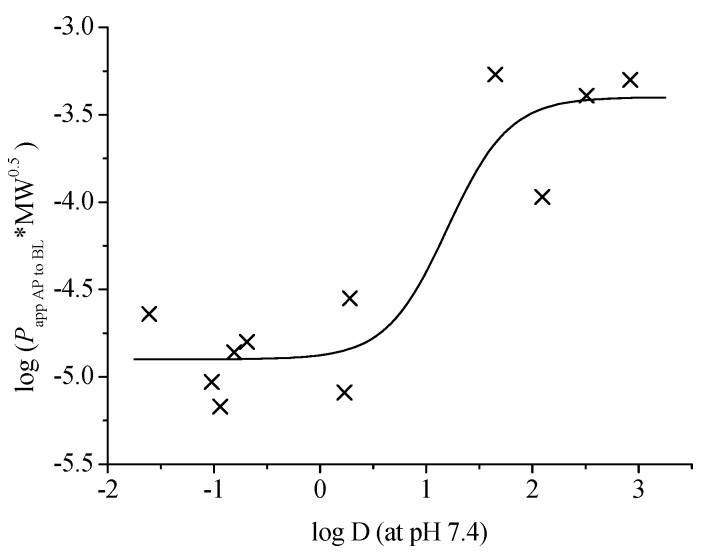
The relationship between the corrected permeability log (*P*_app AP→BL_*MW^0.5^) and log D (at pH 7.4) of the compounds **1**, **3**–**12** of TMF.

Physicochemical characters, such as log P, log D and polar surface area *etc.*, have recently been used to study the relationship between chemical structure and intestinal permeability. Among them, we have found that log D (at pH 7.4) is a simple but potent indicator for the prediction of the biomembrane permeability [20,21]. Here the corrected *P*_app AP→BL_ by molecular weight (MW), log (*P*_app AP→BL_*MW^0.5^) *vs.* log D (at pH 7.4), was again used to investigate whether the potential rule is reproducible for the isoflavonoids and the analogues in TMF. A similar sigmoid dependency between the log D (at pH 7.4) and the corrected log *P*_app_ of the eleven compounds was found (Figure 4). All isoflavone glycosides (**1**, **3**–**7**) with low log D values (–1.61 to 0.23), indicating hydrophilic compounds, clustered within the region of low permeability. While the log D values of the isoflavone aglycones **10**–**12** are 1.65 to 2.92, the curve reached a plateau, implying good permeabilities. The results further confirmed a hypothesis that only medium lipophilicity with a log D range of about 1.5–4.0 can result in a good permeability, and both excessive hydrophilicity and lipophilicity will lead to a weak permeability. The other two puerol derivatives **8** and **9** were roughly in line with the trend of the curve. It seems that a single log D may be more precise and predictable for derivatives of the same skeleton.

## 3. Experimental Section

### 3.1. Chemicals, Materials and Instruments

Methanol (MeOH) and acetonitrile (MeCN) were of HPLC-grade from J.T. Baker (Center Valley, PA, USA). HPLC-grade formic acid was from Dikma Tech. Inc. (Beijing, China). Deionized water (H_2_O) was obtained from a Milli-Q Ultra-pure water system (Millipore, Bedford, MA, USA). Naringin was purchased from the Tianjin Jianfeng Natural Product R & D Co. Ltd. (Tianjin, China). Reference standards of 3′-hydroxypuerarin (**1**), protocatechuic aldehyde (**2**), 3′-hydroxymirificin (**3**), puerarin (**4**), 3′-methoxypuerarin (**5**), mirificin (**6**), daidzin (**7**), (±)-puerol B-2"-*O*-glucopyranoside (**8**), puerol A (**9**), daidzein (**10**), genistein (**11**), formononetin (**12**) (structures are shown in Figure 1) were prepared from TMF and their chemical structures were determined by UV, mass and nuclear magnetic resonance spectra in our group [4,13]. The purities of all reference standards were above 98% by HPLC analysis.

Tryptone, beef extract, beef liver extract powder, digestibility serum powder, and proteose peptone were purchased from Beijing Shuangxuan Microorganism Medium Product Factory (Beijing, China). Sodium thioglycolate, l-cysteine hydrochloride, and glucose were purchased from Sigma Chemical Co. (Deisenhofen, Germany). Yeast extract was obtained from Unipath Ltd. (Basingstoke, Hampshire, UK).

The crude drugs of Puerariae Lobatae Radix, Salviae Miltiorrhizae Radix and Chuanxiong Rhizoma were obtained from Shenwei Medicine Co., Ltd. (Shijiazhuang, Hebei, China). They were identified as roots of *Pueraria lobata* (Willd.) Ohwi, roots of *Salvia miltiorrhiza* Bge. and rhizomes of *Ligusticum chuanxiong* Hort., respectively, by Professor Xiu-Wei Yang of School of Pharmaceutical Sciences of Peking University (Beijing, China). The voucher specimens have been deposited in Department of Natural Medicines, School of Pharmaceutical Sciences, Peking University (Beijing, China). To prepare TMF extracts for the biotransformation by HIF, the three TCMs were mixed together at the same proportion and then extracted with boiling water twice (first for 1.5 h and second for 1 h). The extract solution was concentrated in the relative density range of 1.18–1.22 and then precipitated with 65% aqueous ethanol for 24 h. The 65% ethanol-soluble part was subsequently filtered, concentrated, and freeze-dried. The freeze-dried powder of TMF was stored at 4 °C before used.

Dulbecco’s Modified Eagle’s Medium (DMEM), Hanks’ Balanced Salts Solution (HBSS), Eagle’s Balanced Salts Solution (EBSS), fetal bovine serum (FBS) and non-essential amino acids (NEAA) were obtained from Gibco Laboratories (Life Science Technologies, Carlsbad, CA, USA). Trypsin, ethylenediamine tetraacetic acid (EDTA), atenolol, propranolol, 2-(4-morpholino)ethanesulfonic acid (MES), dimethyl sulfoxide (DMSO) were purchased from Sigma-Aldrich (St. Louis, MO, USA), and the purities of propranolol and atenolol were above 98%. Penicillin and streptomycin were purchased from Huabei Pharmaceutical Group Co., Ltd. (Shijiazhuang, Hebei, China). *p*-Nitrophenylphosphate disodium salt hexahydrate (*p*-NPP) was purchased from Amresco, Inc. (Solon, OH, USA). Alkaline phosphatase (AKPase) kit was purchased from Zhong Sheng Bei Kong Bio-Technology and Science Inc. (Beijing, China). Other chemicals were of analytical grade and solvents used in HPLC were of HPLC grade. Transwell™ plates of 12 wells (insert diameter 12 mm, pore size 3.0 μm, insert membrane growth area 1.12 cm^2^) were purchased from Corning Inc. (Cambridge, MA, USA).

For HPLC quantitative analysis, a Ultimate 3000 chromatographic system (Dionex, Germering, Germany) including a pump, an autosampler, a column compartment, a diode array detector, and a Chromeleon Workstation (version 6.80) was used. Chromatographic separation was performed on a Diamonsil™ C_18_ column (250 mm × 4.6 mm, 5 μm, Dikma, Beijing, China) equipped with a C_18_ guard column cartridge system (8 mm × 4 mm, 5 μm; Dikma).

A Thermo Scientific 1029 Forma Anaerobic System (Forma Scientific, Inc., Marietta, OH, USA) was applied to create anaerobic conditions and Oxoid BR0055 anaerobic indicator was used. A mode HZ-2111K-B shaking incubator (Hualida Laboratory Equipment Company, Jiangsu, China) was used for biological sample incubation.

### 3.2. Preparation of GAM and HIF

The general anaerobic medium (GAM) of HIF was prepared as follows: 10.0 g of tryptone, 10.0 g of proteose peptone, 13.5 g of digested serum powder, 5.0 g of yeast extract, 2.2 g of beef extract, 1.2 g of beef liver extract powder, 3.0 g of glucose, 2.5 g of KH_2_PO_4_, 3.0 g of NaCl, 5.0 g of soluble starch, 0.3 g of l-cysteine hydrochloride, and 0.3 g of sodium thioglycolate were dissolved in H_2_O. Adjust the pH of GAM solution to 7.1–7.2 with 1M NaOH aqueous solution and supply the total volume to 1 L. Ten grams of fresh feces obtained from a Chinese healthy male volunteer, who had not taken any medicine in three months, were immediately homogenized and suspended in GAM (50 mL) solution. The HIF was undertaken according to the previous paper of our laboratory [22]. Preparation of the biotransformation products of TMF by HIF was followed by the method of previous research [23].

### 3.3. Time Course the Twelve Constituents of TMF by HIF

A biotransformation system consisting of HIF (10 mL) and TMF (10 mg) was anaerobically incubated at 37 °C. All experiments were carried out in sextuplicate at each time point. The biotransformation reaction was stopped at 0.5, 1, 2, 4, 8, 12, 24, 36, 48, 60 h. The biotransformation solution was extracted with *n*-butanol (each 10 mL) three times. The combined *n*-butanol layer was evaporated to dryness under a gentle stream of nitrogen. MeOH (400 μL) was used to dissolve the residue which acted as a deproteinization agent. The resultant solution was vortex-mixed for 1 min and centrifuged at 16,000 *g* for 10 min at 4 °C. The supernatant was collected and filtered through a 0.22 μm filter as the test solution. The blank sample was prepared using TMF-free medium solution with the same processing steps. The test and blank solutions were analyzed by HPLC using a gradient mobile phase consisting of solvent A (MeCN) and B (0.4% acetic acid aqueous solution) using a gradient elution of 11%–12% A at 0–28 min, 12%–15% A at 28–30 min, 15%–17% A at 30–45 min, 17%–22% A at 45–65 min, 22%–28% A at 65–66 min, 28%–35% A at 66–85 min, 35%–50% A at 85–86 min and 50% A at 86–98 min. The column temperature was set at 30 °C and detection wavelength was set at 254 nm.

### 3.4. Cell Culture

Human colon adenocarcinoma cell line Caco-2 (ATCC #HTB-37) was purchased from American Type Culture Collection (ATCC, Rockville, MD, USA). The standardized conditions and cell viability assay of Caco-2 cell culture were undertaken according to the standard operating protocols [17]. Briefly, the Caco-2 cells were cultured in DMEM containing d-glucose (4.5 g/L), NaHCO_3_ (3.7 g/L), supplemented with 10% FBS, 1% NEAA, penicillin (100 U/mL), and streptomycin (100 μg/mL) in an atmosphere of 5% CO_2_ and 95% relative humidity at 37 °C. All cells were between passages 45 and 60.

### 3.5. Caco-2 Permeation Experiment of the Eleven Compounds

The Caco-2 cells were seeded at a density of 1.0 × 10^5^ cells/mL on a 12-wells Transwell™ insert filter and left to grow for 21 d to reach confluence and differentiation. Differentiation of Caco-2 cells was assayed by determining the activity of alkaline phosphatase with an assay equipment on the 3rd and 14th days [24] and detecting the cellular morphology by transmission electron microscopy on the 21st day [17]. The integrity and transportation ability of the Caco-2 cell monolayer were examined by measuring the transepithelial electrical resistance (TEER) with an epithelial voltohmmeter (EVOM, World Precision Instrument, Sarasota, FL, USA) before and after the transport. Only cell monolayers with TEER values above 500 Ω·cm^2^ were qualified for the transport assays. Standard compounds, propranolol and atenolol were run as the active and passive transport marker, respectively [25,26].

Ten mM stock solutions of test compounds were prepared in DMSO and diluted with HBSS to 50 μM before transport experiments. After washing the Caco-2 cell monolayer twice with prewarmed HBSS medium (pH 7.4), the transport experiments were carried out by adding the test samples to either the apical (AP, 0.5 mL) or basolateral side (BL, 1.5 mL) while the receiving chamber contained the corresponding volume of HBSS medium. After shaking at 55 rpm for 1 h at 37 °C in a water bath, samples were collected from both sides of Caco-2 cell monolayer and immediately frozen, lyophilized and preserved below −20 °C for subsequent HPLC analysis.

To determine the eleven compounds, the lyophilized samples from both sides of the Transwell were dissolved in 200 μL of MeOH and centrifuged at 15,000 *g* for 10 min. Twenty μL aliquot of the supernatant solution was used for HPLC analysis. The cells were extracted after transport assays with 300 μL of MeOH, which was used to measure the amount of the intracellular accumulation for the test compounds. The HPLC mobile phase consisted of solvent A (MeCN) and B (0.4% acetic acid aqueous solution) using isocratic elutions (Supplementary Materials Table S3).

In the meantime, the apparent permeability coefficients, *P*_app_ (cm/s) were calculated on the basis of the following equation:

*P*_app_ = (Δ*Q*/Δ*t*)/(*AC*_0_) (1)

where Δ*Q*/Δ*t* is the appearance rate of the test compound on the receiver side (μmol/s); *A* is the membrane surface area (cm^2^); *C*_0_ is the initial concentration of the test compound at the donor side (μM). All the experiments were conducted in triplicate and the data were expressed as mean ± S.D.

## 4. Conclusions

The TMF biotransformation results showed that its glycoside compounds were converted into the corresponding aglycones (isoflavone and puerol) and as time went by, the relative content of aglycones obviously increased. Daidzein (**10**), the principal isoflavone aglycone in TMF, has been marketed as a prescription drug in the treatment of hypertension, coronary heart disease, and cerebral thrombosis in China. Accordingly we predict that the aglycone compounds might be the real active ingredients in TMF, and a permeability test was needed to determine the intestinal absorption capacity of the compounds. From the permeability result, isoflavone aglycones were well-absorbed compounds, which was consistent with the previous result [27]. In addition, the intestinal absorption capacity of puerol aglycones was better than puerol glycosides, and the methylation reaction could promote the intestinal absorption capacity of isoflavone glycosides. In conclusion, the constituents of TMF can be transformed by HIF, and the intestinal absorption capacities of biotransformation products were better than the original compounds. The methodology in this paper will be useful for studying the TCMs and clarifying the molecular basis of therapeutics.

## Supplementary Materials

HPLC method validations are available as Supplementary Materials, which may be accessed at: http:// www.mdpi.com/1420-3049/20/10/18704/s1.
