# Supplementary material for: Study of the Biotransformation of Tongmai Formula by Human Intestinal Flora and Its Intestinal Permeability across the Caco-2 Cell Monolayer
Source: Molecules. 2015 Oct 15;20(10):18704–16. doi: 10.3390/molecules201018704 (PMC6331934; doi:10.3390/molecules201018704)
Supplement: Supplementary file 1 [file molecules-20-18704-s001.pdf]

Supplementary Materials

1. HPLC Method Validation for Quantification of Twelve Constituents of TMF in the Time Course Experiments

1.1. Preparation of Stock Solutions, Calibration Samples, and QC Samples

The stock solutions of twelve constituents of TMF and naringin as an internal standard (I.S.) were separately prepared by dissolving the constituents in 65% MeOH aqueous to the final concentration and kept at 4 °C. To construct the calibration curves, the working solutions were prepared by appropriate dilution of the stock solution with blank heat-inactivated HIF incubation solution. Quality control (QC) samples of the mixed analytes in heat-inactivated HIF solution were prepared at low, medium and high concentrations.

1.2. Specificity, Linear Range, Lower Limits of Detection and Quantification

Specificity was established by confirming the lack of interfering peaks at the retention times for twelve constituents of TMF and I.S. The calibration curve was constructed by plotting peak area ratios of twelve constituents of TMF and I.S. versus the corresponding concentration to six concentration levels. The lower limit of detection (LLOD) and lower limit of quantification (LLOQ) were set at the lowest concentration giving a signal-to-noise ratio of 3:1 and 10:1, respectively (Table S1).

1.3. Precision, Accuracy, Stability and Recovery

The intra-day and inter-day precision and accuracy were evaluated by assaying the low, medium, and high concentrations of QC samples containing I.S. solution. The intra-day variation was determined by assaying three replicates on the same day and inter-day variation was assayed for three consecutive days. The precision was expressed as the means and relative standard deviations (RSD). The accuracy was assessed by comparing the calculated concentration to the theoretical concentration. Stability of twelve constituents of TMF in the heat-inactivated HIF incubation solution during the storage at room temperature was assessed by analyzing QC samples after 24 h. The results were compared with freshly prepared QC samples. The extraction recoveries were determined at three concentrations of QC samples by comparing peak areas extracted from the heat-inactivated HIF incubation solution with those of the mean peak areas of standard solutions in MeOH (Table S2).

2. HPLC Method Validation for the Eleven Compounds of TMF in the Caco-2 Permeation Experiments

2.1. Linear Equations, Precision and Accuracy

The HPLC mobile phase consisted of solvent A (MeCN) and B (0.4% acetic acid aqueous solution) using isocratic elutions (Table S3). The column temperature was set at 30 °C and detection wavelength was set at 254 nm. Peak area measurement was used to obtain calibration curves of each biotransformation product. The calibration curves were constructed by plotting peak area (y, mAU*min) versus concentration (x, μM). The linear equations and precision of the eleven compounds of TMF in the Caco-2 permeation experiment were shown in Tables S3 and S4.

Table S1. The calibration curve, correlation coefficient and linear range, LLOD, and LLOQ of the twelve constituents of TMF in the time course experiments.

Compound	Calibration Curve	*r*	Linear Range (μg/mL)	LLOD (μg/mL)	LLOQ (μg/mL)
3′-hydroxypuerarin (**1**)	$y = 0.0149\,x + 0.0016$	0.9972	0.33–84.57	0.03	0.08
protocatechuic aldehyde (**2**)	$y = 0.0099\,x + 0.0079$	0.9970	0.57–146.36	0.02	0.07
3′-hydroxymirificin (**3**)	$y = 0.0073\,x + 0.0043$	0.9991	0.63–160.09	0.03	0.08
puerarin (**4**)	$y = 0.0127\,x - 0.0857$	0.9980	8.37–2142.58	0.35	1.05
3′-methoxypuerarin (**5**)	$y = 0.0062\,x + 0.0027$	0.9994	2.79–715.31	0.12	0.35
mirificin (**6**)	$y = 0.0115\,x + 0.0029$	0.9992	0.78–200.16	0.03	0.1
daidzin (**7**)	$y = 0.0154\,x + 0.0021$	0.9991	3.02–772.39	0.13	0.38
(±)-puerol B-2″-*O*-glucopyranoside (**8**)	$y = 0.0023\,x - 0.0006$	0.9968	7.84–2006.37	0.65	1.96
puerol A (**9**)	$y = 0.0044\,x + 0.0079$	0.9985	1.13–288.04	0.05	0.14
daidzein (**10**)	$y = 0.0131\,x + 0.0280$	0.9958	2.47–632.40	0.1	0.31
genistein (**11**)	$y = 0.0156\,x + 0.0073$	0.9961	1.17–300.13	0.05	0.15
formononetin (**12**)	$y = 0.0099\,x + 0.0085$	0.9941	0.99–254.13	0.04	0.12

Table S2. The accuracy and precision, extraction recovery, stability of the twelve constituents of TMF in the time course experiments.

Compound	Intra-Day		Inter-Day		Recovery		Stability	
	RSD (%)	Accuracy (%)	RSD (%)	Accuracy (%)	Mean (%)	RSD (%)	Accuracy (%)	RSD (%)
3′-hydroxypuerarin (**1**)	7.60	105.31	11.14	106.07	62.80 ± 6.13	11.80	106.26	8.14
	10.77	93.95	8.77	94.60	75.65 ± 1.29	7.06	97.15	9.42
	7.99	99.75	4.24	100.24	78.13 ± 0.25	10.31	103.57	6.00
protocatechuic aldehyde (**2**)	4.78	100.23	4.48	96.46	78.04 ± 7.26	13.19	99.75	6.09
	9.91	100.94	7.99	99.25	72.52 ± 0.51	7.40	101.50	9.40
	12.67	97.98	3.31	100.57	73.75 ± 0.17	13.24	104.81	8.62
3′-hydroxymirificin (**3**)	9.83	108.39	11.88	104.40	84.99 ± 2.04	5.38	101.85	8.80
	9.74	105.87	9.50	105.93	81.89 ± 0.48	9.22	103.75	8.99
	10.21	99.65	6.36	92.32	68.91 ± 0.09	12.21	102.28	7.99

Table 2. *Cont.*

Compound	Intra-Day		Inter-Day		Recovery		Stability	
	RSD (%)	Accuracy (%)	RSD (%)	Accuracy (%)	Mean (%)	RSD (%)	Accuracy (%)	RSD (%)
puerarin (**4**)	5.80	102.83	5.65	102.87	71.07 ± 17.40	2.40	102.99	7.59
	10.56	93.17	7.09	93.32	84.21 ± 5.36	3.78	104.06	10.15
	10.89	103.73	6.21	94.70	82.75 ± 2.15	13.05	103.94	6.97
3′-methoxypuerarin (**5**)	8.98	106.57	4.66	105.66	78.81 ± 5.54	4.22	105.66	10.78
	11.52	93.71	6.95	95.08	84.00 ± 0.94	3.32	104.86	6.98
	11.44	109.54	7.69	100.32	86.65 ± 0.09	2.77	105.51	2.75
mirificin (**6**)	9.49	102.40	9.85	102.93	71.40 ± 6.38	10.31	104.27	7.45
	9.25	94.83	7.38	95.17	75.06 ± 0.75	5.55	100.65	11.06
	6.78	99.00	7.47	92.79	81.47 ± 0.10	6.80	101.96	7.13
daidzin (**7**)	3.39	104.11	7.70	101.82	76.15 ± 11.59	3.41	106.16	5.99
	7.92	90.08	8.91	92.62	85.74 ± 6.45	10.09	105.50	8.27
	10.27	93.62	10.43	93.16	84.05 ± 0.63	9.12	97.96	9.90
(±)-puerol B-2″-*O*-glucopyranoside (**8**)	10.05	100.27	10.78	106.66	86.95 ± 8.02	4.76	104.69	10.35
	7.77	96.81	11.20	97.91	80.32 ± 1.10	4.76	103.81	12.52
	11.78	102.20	6.73	98.32	72.70 ± 0.20	7.11	104.03	7.66
puerol A (**9**)	8.63	101.09	9.01	101.23	78.73 ± 3.13	7.50	102.79	9.18
	11.87	98.82	9.94	96.11	78.46 ± 0.44	6.27	98.42	12.03
	8.04	102.78	9.52	91.29	70.50 ± 0.10	10.51	101.60	5.56
daidzein (**10**)	9.06	100.30	4.52	99.14	85.17 ± 13.37	4.26	104.15	10.13
	8.11	92.74	4.05	95.91	83.25 ± 3.43	8.18	97.92	12.77
	11.42	96.85	3.05	94.28	76.61 ± 0.37	7.76	97.21	5.06
genistein (**11**)	7.24	96.62	3.13	101.29	84.04 ±17.29	10.76	103.62	10.23
	11.28	93.86	9.49	95.35	72.48 ± 1.41	5.89	99.35	12.29
	8.72	94.10	5.87	92.29	82.88 ± 0.21	7.22	101.80	11.82
formononetin (**12**)	6.95	101.04	8.49	100.58	81.94 ± 4.73	5.57	101.18	9.76
	10.00	94.50	7.65	100.43	77.15 ± 1.39	11.29	103.35	9.71
	11.68	100.33	6.38	98.80	78.78 ± 0.14	6.08	99.84	10.66

Table S3. The mobile phase, the retention time and linear equation of the eleven compounds of TMF in the Caco-2 permeation experiments.

Compound	Proportion of Mobile Phase *	t_R/min	Regression Equation	*r*
3′-hydroxypuerarin (**1**)	35:65:0.3	6.235	$y = 0.3088x - 0.1296$	1.0000
3′-hydroxymirificin (**3**)	35:65:0.3	6.656	$y = 0.2593x - 0.5262$	0.9998
puerarin (**4**)	35:65:0.3	8.569	$y = 0.4013x - 0.3359$	1.0000
3′-methoxypuerarin (**5**)	35:65:0.3	10.501	$y = 0.3789x + 0.1188$	0.9999
mirificin (**6**)	38:62:0.3	7.564	$y = 0.3920x - 0.0305$	1.0000
daidzin (**7**)	44:56:0.3	6.466	$y = 0.3540x - 0.2544$	0.9999
(±)-puerol B-2″-*O*-glucopyranoside (**8**)	30:70:0.4	6.880	$y = 0.0364x + 0.0685$	0.9988
puerol A (**9**)	35:65:0.4	7.547	$y = 0.0015x + 0.0016$	0.9987
daidzein (**10**)	60:40:0.3	8.373	$y = 0.3610x - 0.7784$	0.9999
genistein (**11**)	50:50:0.4	6.580	$y = 0.5530x + 0.2951$	0.9999
formononetin (**12**)	72:28:0.3	8.218	$y = 0.3840x + 0.1559$	1.0000

* The mobile phase consisted of MeCN, H_2O, and acetic acid in turn.

Table S4. The accuracy and precision of the eleven compounds of TMF in the Caco-2 permeation experiments.

Compound	Intra-Day		Inter-Day	
	Precision RSD (%)	Accuracy (%)	Precision RSD (%)	Accuracy (%)
3′-hydroxypuerarin (**1**)	1.40	100.16	3.08	99.14
	1.17	98.20	3.93	95.73
	2.34	98.92	5.04	101.36
3′-hydroxymirificin (**3**)	0.92	98.19	0.68	98.03
	1.91	98.97	2.52	97.89
	1.17	117.16	2.28	117.93
puerarin (**4**)	1.60	99.71	2.35	97.57
	1.70	98.68	1.98	104.22
	1.48	99.58	1.59	103.96
3′-methoxypuerarin (**5**)	1.44	101.49	2.96	100.43
	0.61	101.17	2.09	102.00
	1.75	103.14	1.26	102.31
mirificin (**6**)	0.97	100.00	2.27	97.46
	1.31	100.59	3.42	100.25
	1.15	99.46	2.98	96.46
daidzin (**7**)	2.32	98.87	3.45	99.68
	2.47	99.71	3.34	103.49
	2.03	101.46	3.82	104.71
(±)-puerol B-2″-*O*-glucopyranoside (**8**)	4.34	104.18	4.41	97.26
	3.60	105.90	5.47	105.36
	2.74	101.17	3.59	98.78

Table 4. *Cont.*

Compound	Intra-Day		Inter-Day	
	**Precision RSD (%)**	**Accuracy (%)**	**Precision RSD (%)**	**Accuracy (%)**
puerol A (**9**)	5.04	95.93	3.89	97.93
	2.78	101.83	5.74	103.61
	4.97	95.99	8.69	104.67
daidzein (**10**)	2.23	97.89	3.30	98.82
	2.92	96.41	3.07	95.99
	0.49	94.82	4.09	97.88
genistein (**11**)	4.55	99.78	4.89	105.51
	3.88	98.54	2.99	100.41
	3.77	101.96	2.20	102.13
formononetin (**12**)	1.49	100.89	1.13	101.15
	0.61	100.71	1.10	99.84
	0.57	100.06	1.01	99.93

2.2. Stability of the Eleven Compounds of TMF in the Transport Medium HBSS

The stability of eleven compounds of TMF was evaluated as the rate of reappearance, which is the percentage of the intact forms remained after incubation at 37 °C in HBSS for 1 h. All compounds tested were stable in pH 7.4 HBSS (>95%)
